# An Autoregressive-Based Motor Current Signature Analysis Approach for Fault Diagnosis of Electric Motor-Driven Mechanisms

**DOI:** 10.3390/s25041130

**Published:** 2025-02-13

**Authors:** Roberto Diversi, Alice Lenzi, Nicolò Speciale, Matteo Barbieri

**Affiliations:** Department of Electrical, Electronic and Information Engineering, University of Bologna, Viale del Risorgimento 2, 40136 Bologna, Italy; alice.lenzi4@unibo.it (A.L.); nicolo.speciale@unibo.it (N.S.); matteo.barbieri15@unibo.it (M.B.)

**Keywords:** condition monitoring, fault diagnosis, electric motor-driven mechanisms, motor current signature analysis, data-driven methods, autoregressive modeling, autoregressive spectral estimation, discrete wavelet transform

## Abstract

Maintenance strategies such as condition-based maintenance and predictive maintenance of machines have gained importance in industrial automation firms as key concepts in Industry 4.0. As a result, online condition monitoring of electromechanical systems has become a crucial task in many industrial applications. Motor current signature analysis (MCSA) is an interesting noninvasive alternative to vibration analysis for the condition monitoring and fault diagnosis of mechanical systems driven by electric motors. The MCSA approach is based on the premise that faults in the mechanical load driven by the motor manifest as changes in the motor’s current behavior. This paper presents a novel data-driven, MCSA-based CM approach that exploits autoregressive (AR) spectral estimation. A multiresolution analysis of the raw motor currents is first performed using the discrete wavelet transform with Daubechies filters, enabling the separation of noise, disturbances, and variable torque effects from the current signals. AR spectral estimation is then applied to selected wavelet details to extract relevant features for fault diagnosis. In particular, a reference AR power spectral density (PSD) is estimated using data collected under healthy conditions. The AR PSD is then continuously or periodically updated with new data frames and compared to the reference PSD through the Symmetric Itakura–Saito spectral distance (SISSD). The SISSD, which serves as the health indicator, has proven capable of detecting fault occurrences through changes in the AR spectrum. The proposed procedure is tested on real data from two different scenarios: (i) an experimental in-house setup where data are collected during the execution of electric cam motion tasks (imbalance faults are emulated); (ii) the Korea Advanced Institute of Science and Technology testbed, whose data set is publicly available (bearing faults are considered). The results demonstrate the effectiveness of the method in both fault detection and isolation. In particular, the proposed health indicator exhibits strong detection capabilities, as its values under fault conditions exceed those under healthy conditions by one order of magnitude.

## 1. Introduction

The reliability and efficiency of machinery are of paramount importance in modern industry. Maintenance strategies such as condition-based maintenance (CBM) and predictive maintenance (PM) have therefore gained increasing importance, especially with the introduction of Industry 4.0 [1,2,3,4]. As a result, online condition monitoring and fault diagnosis (FD) of electromechanical systems have become crucial tasks in many industrial applications. Condition monitoring, upon which CBM and PM are based, involves tracking the system state of health to detect fault occurrences [2,5,6]. Indeed, early detection and identification of faults allow minimizing downtime, optimizing maintenance schedules, and preventing costly repairs.

Any condition monitoring (CM) procedure requires sensor measurements and analysis of signals (such as vibration, speed, current, and temperature) that are relevant to the performance state of the system’s components. Although vibration analysis remains the most widely used method [5,7,8], motor current signature analysis (MCSA) has gained increasing attention in recent years [9,10,11,12,13,14,15,16,17]. MCSA is a non-intrusive and cost-effective approach when compared to vibration-based techniques, as it relies only on the motor phase currents, which are already used in motor control. Other advantages over vibration analysis include simpler sensor installation, reduced sensitivity to sensor installation location, and less sensitivity to external factors such as background noise. Initially, this technique was adopted only to monitor electric motor components [18,19] (e.g., windings, rotor, bearings, resistance); this allows the tracking of the motor’s internal state of health, but it does not provide insight into the condition of the mechanical systems driven by the motor. In the last years, MCSA has also been applied to fault diagnosis of components in the mechanisms attached to the electric motor, such as gears [9,11,13], bearings [12,13], belt conveyor systems [16], and rotate vector reducers [15]. In fact, faults in the mechanical load driven by the motor, such as bearing defects, gear misalignments, and unbalanced loads, manifest as changes in the motor’s current behavior [9,10,12].

To extract features for FD from the raw current signals frequency domain, time–frequency domain, or combinations of time and frequency domain techniques have been proposed in the literature. In [9], the discrete wavelet transform (DWT) is combined with fast Fourier transform (FFT) to trace the sidebands of the gear mesh frequencies (GMF). In [10], amplitude demodulation and frequency demodulation are applied to the current drawn by the induction motor to detect the rotating shaft frequencies and GMFs. Then, DWT is applied for denoising. The effects induced by gear tooth surface damage faults on the torque oscillation profile are investigated in [11]. In particular, it is shown that such effects produce fault-related frequencies in the stator current spectrum and specific harmonics as integer multiples of the rotation frequency in the stator current space vector instantaneous frequency spectrum. In [12], an efficient method is presented for extracting defective bearing characteristics from the stator current of a loaded machine using the continuous wavelet transform (CWT) based on Morlet’s complex wavelet. In particular, a feature extraction technique is proposed to cope with the fact that the peaks in the frequency spectrum corresponding to the fault components have very low amplitudes and are usually obscured by noise. For this reason, 2D and 3D scalograms of stator current signatures for both healthy and damaged bearings were used to characterize the defects in the time–frequency domain. A health indicator that exploits both a time domain feature (peak-to-peak values) and a frequency domain feature (maximum value of the FFT) is proposed in [13]. The obtained indicator is then used to classify gear and bearing faults through an adaptive neuro-fuzzy inference system. In [14], data acquired from multiple current sensors are fused using a 2D convolutional neural network (CNN), with features extracted from the FFT spectra of the current signals. The CNN is used to classify different types of gear faults.

This paper proposes a method based on autoregressive (AR) spectral estimation of wavelet-processed motor currents. Autoregressive modeling is a very popular tool for time series analysis and spectral estimation [20,21,22]. It is well known that AR models provide more accurate spectral estimates with respect to classical Fourier-transform-based methods, like periodograms and the Blackman–Tukey method. This is mainly due to the fact that, unlike FFT-based methods, the estimated AR spectra (power spectral densities) do not have the sin(x)/x transform response characteristic of conventional windowed spectra and therefore, they do not suffer from sidelobe leakage and spectral smearing. Moreover, the AR power spectral density (PSD) has been shown to be highly sensitive to system changes, making it particularly effective for fault diagnosis applications [23,24,25,26,27,28,29,30]. The AR PSD procedure to perform online condition monitoring requires first estimating a nominal AR model from data gathered under healthy conditions and its PSD. Afterwards, online condition monitoring is achieved by continuously or periodically estimating the AR model from the online data, as well as its PSD, and eventual departures from the nominal AR PSD are detected through several possible methods, in our case the symmetric Itakura–Saito distance, also referred to as COSH distance. An alternative procedure to quantify the dissimilarity between the current and nominal behavior could involve the Fourier spectra of the motor currents; however, this diagnostic process presents more difficulties compared to AR-based methods [23,24,26,27]. In cases where Fourier-based methods offered fault detection, the robustness of the detection thresholds was more critical than in the AR case, since the spectral distances were smaller [26,27].

As mentioned above, AR modeling is not applied directly to the raw current signals. Instead, a multiresolution analysis (MRA) of the motor currents is first performed using the discrete wavelet transform with Daubechies filters [31,32]. Each current is decomposed into approximation and detail components. In particular, suitable levels are selected based on the approximate entropy criterion, and the associated detail components are the signals to which AR spectral estimation is applied. The MRA preprocessing phase enables the separation of noise, disturbances, and variable torque effects from the current signals. Consequently, the effects of faults are amplified, and more robust detection thresholds can be defined. In summary, the entire procedure combines the useful properties of the discrete wavelet transform (DWT) with the aforementioned advantages of AR spectral estimation. It is worth highlighting that the proposed method belongs to the so-called data-driven fault diagnosis approaches [4,5,33,34], as it does not rely on any physical knowledge of the electromechanical systems to be monitored. This also provides great versatility with regard to the type of components to be diagnosed (e.g., bearings, gears, shafts).

The proposed method has been validated by considering the effects of two very different situations on motor currents: unbalanced load and bearing failure. For the first case, real data were collected while performing electric cam movement tasks in a laboratory experiment. This is a very common situation, as many industrial machines rely on electric cams to perform complex tasks that require synchronization between the various mechanisms involved [35]. In the second case, data were obtained from a public domain database [36]. The findings obtained from both scenarios substantiate the efficacy of the method in detecting and isolating faults. It is worth highlighting that the proposed approach differs from those previously described as it does not reference a specific machine component or fault type. Moreover, the parameters required to determine the reference model are computed by using only data collected under healthy conditions. This is a very interesting aspect when fault diagnosis procedures are performed in real industrial contexts, where a large amount of data are collected during normal operating conditions, but very few data related to specific faulty conditions are available.

The remainder of the paper is structured as follows. The proposed fault diagnosis procedure is described in Section 2. In particular, the whole procedure is first outlined. The multiresolution analysis of the raw current signals performed using a discrete wavelet transform is then described in Section 2.1 and Section 2.2 shows how to determine a health indicator by applying AR spectral estimation to the selected wavelet details. Section 3 describes the testbeds on which the proposed approach was tested and discusses the obtained results. Section 4 concludes the paper.

The nomenclature for the relevant variables used in the paper is provided in Table 1.

## 2. Fault Diagnosis Procedure

**Figure 1:**
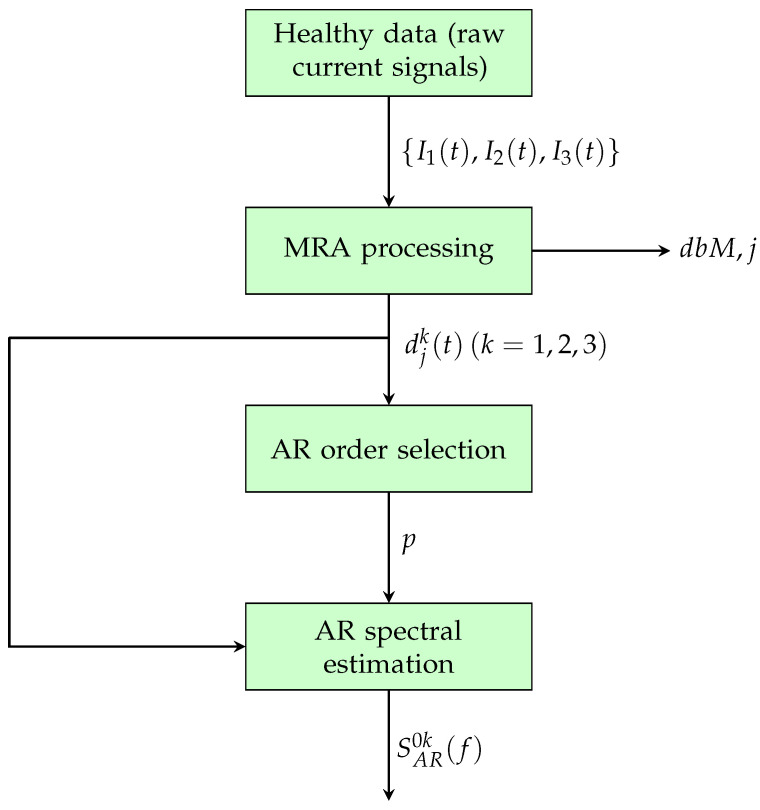
Parameter setting phase of the FD procedure.

**Figure 2:**
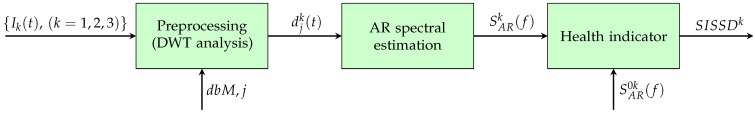
Online condition monitoring phase of the FD procedure.

The fault diagnosis (FD) procedure proposed in this paper consists of the two phases depicted in Figure 1 (parameter setting) and Figure 2 (online condition monitoring), performed on (possibly overlapped) data segments.

The first phase (parameter setting) is performed by using only current data collected under normal (healthy) operating conditions. It consists of the following steps:

- A multiresolution analysis of the three-phase motor healthy current signals $I_{1}\left(t\right)$, $I_{2}\left(t\right)$, $I_{3}\left(t\right)$ is performed using the discrete wavelet transform (DWT). Each current is then decomposed into approximation and detail components. A suitable wavelet type dbM and detail level *j* are selected based on the approximate entropy criterion. The associated details $d_{j}^{k}\left(t\right),\;\left(k=1,2,3\right)$ are the signals from which features are extracted for fault diagnosis. In particular, the use of the details allows for the separation of noise, disturbances, and variable torque effects from the current signal, thereby amplifying fault-induced system changes.
- Suitable features for fault diagnosis are extracted through AR modeling of the selected details. A proper AR order *p* is first selected by means of some model selection criteria. Then, reference AR models are estimated, and the corresponding reference AR power spectral densities (PSDs) $S_{AR}^{0k}\left(f\right),\;\left(k=1,2,3\right)$ are computed from the AR coefficients. This leads to a very accurate estimation of the PSD of the details, which can be used to define a health indicator representing the current state of the mechanical system.

The second phase (online condition monitoring) is performed during online working conditions. The measured data are segmented into (possibly overlapped) frames. For each frame, the steps are as follows:

- A DWT preprocessing is performed on the motor currents by using the wavelet type dbM and level detail *j* selected in the parameter setting phase, to obtain the detail signals $d_{j}^{k}\left(t\right),\;\left(k=1,2,3\right)$.
- An AR model is estimated for each detail signal $d_{j}^{k}\left(t\right)$, using the previously selected order *p*, and the associated AR PSD $S_{AR}^{k}\left(f\right)$ is computed.
- Each computed PSD $S_{AR}^{k}\left(f\right)$ is then compared with the corresponding reference PSD $S_{AR}^{0k}\left(f\right)$ computed in the first phase to check if a system change is occurring. The comparison is made by means of the symmetric Itakura–Saito spectral distance (SISSD). Then, the outputs of the online CM phase are $SISSD^{k},\;\left(k=1,2,3\right)$.

The following subsections describe the aforementioned steps in more detail.

### 2.1. Multiresolution Analysis of Current Signals

This section provides an overview of the first data processing phase, which employs the Wavelet Transform with Daubechies filters [32]. The signal to which multiresolution analysis is applied is denoted by s(t), where $s\left(t\right)\in \{I_{1}\left(t\right),I_{2}\left(t\right),I_{3}\left(t\right)\}$. Once the analysis level *j* and filter order dbM have been selected, the AR model is identified in the extracted signal, as described in Section 2.2.

#### 2.1.1. Multiresolution Analysis

A multiresolution analysis (MRA) is defined as a collection of nested subspaces denoted by $V_{j}$, with j∈Z, which satisfy a set of properties [31,32]. In particular, an MRA involves the successive projection of the signal s(t) to be studied in each of the approximation subspaces $V_{j}$. Since $V_{j}\subset V_{j+1}$, the approximation in $V_{j}$ is coarser than that in $V_{j+1}$. The detail is the information removed when going from one approximation to another. MRA demonstrates that detail signals can be obtained by projecting s(t) onto a set of $W_{j}$ subspaces which are known as wavelet subspaces.

In the context of MRA theory, it is shown that there exists a function ψ(t), designated as the mother wavelet, which serves as a Riesz basis for $W_{j}$ and is derived from the scaling function ϕ(t), which represents the basis for the subspace $V_{j}$. The two functions are solutions of a two-scale difference equation:

(1) $$\begin{array}{cc} \varphi (t) & =\sqrt{2}\sum_{n\in Z}h_{n}\varphi \left(2t-n\right) \end{array}$$

(2) $$\begin{array}{cc} \psi (t) & =\sqrt{2}\sum_{n\in Z}g_{n}\varphi \left(2t-n\right) \end{array}$$

where $g_{n}={\left(-1\right)}^{n}h_{1-n}$ and the sequences ${\left\{h_{n}\right\}}_{n\in Z}$ and ${\left\{g_{n}\right\}}_{n\in Z}$ can be interpreted as the impulse response of a low-pass digital scaling filter and a high-pass wavelet filter, having the same cutoff frequency [31]. The application of these filters to the original signal results in the generation of two new signals. The first of these comprises the lower frequencies, while the second comprises the higher frequencies. An additional pair of filters can reconstruct the original signal if they meet specific complementarity constraints. The design of the sequence ${\left\{h_{n}\right\}}_{n\in Z}$ permits the construction of wavelet systems with desirable properties, including filter regularity, as exemplified by the Daubechies dbM family [37].

When the mother wavelet is real and the signal has finite energy, the discrete wavelet transform (DWT) can be seen as a mapping from $L^{2}\left(R\right)$ to $l^{2}\left(Z\right)$ given by

(3) $$s\left(t\right)⟶\left\{a_{J}\left(t\right),\;t\in Z\right\},\;\;\left\{d_{j}\left(t\right),\;j=1,\ldots ,J,t\in Z\right\}.$$

These coefficients are defined through inner products of the signal with shifted and scaled versions of the scaling function and the mother wavelet, whose definition depends on whether one chooses to use an orthogonal, semi-orthogonal, or bi-orthogonal DWT [37].

For this work, we have chosen Daubechies wavelets dbM, which have compact support and the highest number of evanescent moments for a given support width, making DWT analysis particularly attractive and feasible. However, alternative wavelet families could be considered to obtain the details to be processed by AR analysis, as long as they allow for the implementation of the discrete DWT approach.

The above decomposition scheme (3) can be repeated more than once. At each iteration, the scheme decomposes the approximation resulting from the previous iteration. If the original sampled signal is $s\left(t\right)=a_{0}\left(t\right)$, t=0,…,N−1, then the trend and the detail series, after *J* iterations, are $a_{J}\left(t\right)$ and $d_{j}\left(t\right)$, j=1,…,J,t=0,…,N−1 respectively. Given that MRA is a scale-based additive decomposition, one can write it as follows:

(4) $$s\left(t\right)=a_{0}\left(t\right)=a_{J}\left(t\right)+\sum_{j=1}^{J}d_{j}\left(t\right)$$

In other words, the information conveyed by a signal can be expressed as a set of details at varying resolutions (or scales) and a low-resolution approximation (or trend). In practice, these are computed by a fast recursive algorithm with a low computational cost, as described by Mallat [31]. The MRA framework allows DWT to be executed with only the use of simple digital filters, thereby enabling its use in real-time applications as well.

#### 2.1.2. Filter Order and Detail Level

The most important step of the preprocessing phase shown in Figure 1 is the procedure described in this subsection. It is carried out only once to select the filter and the level of analysis to construct the data set that will later be used to estimate the AR model, as described in Section 2.2.

The first practical challenge faced when undertaking a wavelet analysis is the selection of a wavelet filter among all possible ones. The selection of an appropriate wavelet depends on the specific analysis goal and the properties of the wavelet that are most suitable for achieving that goal. In this particular case, the decision-making process is influenced by two factors. Firstly, the use of very short wavelet filters has the potential to introduce unintended artefacts into the analysis results. Secondly, the application of high-order wavelet filters may result in a reduction in the localization property of DWT coefficients while concurrently increasing the computational burden. Accordingly, a general strategy is to use the smallest order that gives reasonable results.

The goal of approximate entropy (ApEn) is to estimate the randomness of a data set without any prior knowledge of its source [38,39]. This is achieved using a method that does not require any *a-priori* assumptions about the data set under investigation. The ApEn algorithm then assigns a non-negative number ε to a given sequence. In general, larger values of ε correspond to greater apparent randomness, while smaller values are indicative of recognizable repetitive patterns. The approximate entropy expresses the regularity of a time series in several dimensions and contains temporal information. This makes it an interesting tool for monitoring system dynamics in general and for diagnosing current machine conditions.

The time series s(t) to be analyzed is processed using the discrete wavelet transform (3) to obtain the signal representation (4). By processing the details $d_{j}\left(t\right)$ it is possible to separate the effects of disturbances, noise, and variable torque from the currents. In some cases, it may also be more advantageous to use the sum of some details and thus obtain the AR spectral estimate by processing a partial reconstruction of the signal. This allows for the amplification of fault-induced variations, resulting in the generation of signals that are more representative.

### 2.2. Fault Diagnosis Through Autoregressive Spectral Estimation

Once the analysis level *j* and filter order dbM have been selected, suitable features for fault diagnosis are extracted through autoregressive (AR) modeling. The use of AR processes is based on the assumption that each detail signal $y\left(t\right)=d_{j}^{k}\left(t\right),\;k=\left(1,2,3\right)$ is a wide-sense stationary random process, at least for a sufficiently small time interval. In this case, y(t) can be described by the following difference equation:

(5) $$y\left(t\right)+a_{1}\;y\left(t-1\right)+\ldots +a_{p}\;y\left(t-p\right)=w\left(t\right)$$

where $a_{1},a_{2},\cdots ,a_{p}$ are the AR coefficients, w(t) is a white process with variance $\sigma_{w}^{2}$ and *p* is the AR order, i.e., the memory of the process. An AR process can be seen as the output of an all-pole transfer function; in fact, by considering the *z*-transform Y[z],W[z] of y(t) and w(t) we get

(6) $$Y\left[z\right]=\frac{W[z]}{A(z)}=\frac{W(z)}{1+a_{1}\;z^{-1}+\cdots +a_{n}\;z^{-p}}.$$

There are many methods for estimating the AR coefficients $a_{1},\cdots ,a_{p}$ and the noise variance $\sigma_{w}^{2}$ starting from a set of available observations y(1),y(2),⋯,y(L). Among them, it is worth mentioning the least squares (LS) approach and the Burg method [20,21,22]. The former is based on the minimization of the sum of squared prediction errors, while the latter uses estimated reflection coefficients to estimate the AR parameters recursively. In this paper, we opt for the LS approach, which is based on the minimization of the sum of the squared prediction errors J(θ):

(7) $$J\left(\theta \right)=\sum_{t=p+1}^{L}{\left(y\left(t\right)-\varphi^{T}\left(t\right)\theta \right)}^{2}$$

where

(8) $$\begin{array}{c} \varphi \left(t\right)={\left[\;-y(t-1)\;\;-y(t-2)\;\cdots \;-y(t-p)\;\right]}^{T} \end{array}$$

(9) $$\begin{array}{c} \theta ={\left[\;a_{1}\;\;a_{2}\;\cdots \;a_{p}\;\right]}^{T}. \end{array}$$

The solution is given by

(10) $$\hat{\theta }={\left(\sum_{t=p+1}^{N}\varphi \left(t\right)\varphi^{T}\left(t\right)\right)}^{-1}\sum_{t=p+1}^{N}\varphi \left(t\right)y\left(t\right).$$

An estimate of the additive noise variance $\sigma_{w}^{2}$ can be computed as follows:

(11) $${\hat{\sigma }}_{w}^{2}=\frac{1}{N-p}\sum_{t=p+1}^{N}{\left(y\left(t\right)-\varphi^{T}\left(t\right)\hat{\theta }\right)}^{2}=\frac{1}{N-p}J\left(\hat{\theta }\right).$$

One of the main reasons for the popularity of AR models in signal processing is the high-resolution power spectrum estimate that can be obtained from their coefficients [20,21]. The power spectral density (PSD) of an AR process easily follows from (6)

(12) $$S_{AR}\left(f\right)=\frac{\sigma_{w}^{2}}{|A\left(e^{-j2\pi f/f_{s}}\right){|}^{2}}=\frac{\sigma_{w}^{2}}{|1+\sum_{k=1}^{p}a_{k}\;e^{-j2k\pi f/f_{s}}{|}^{2}}$$

and is an approximation of the PSD $S_{y}\left(f\right)$ of the signal y(t), given by the discrete-time Fourier transform of its autocorrelation function $r_{y}\left(\tau \right)$:

(13) $$S_{y}\left(f\right)=\sum_{\tau =-\infty }^{\tau =\infty }r_{y}\left(\tau \right)\;e^{-j2\pi \tau f/f_{s}}$$

where $r_{y}\left(\tau \right)=E\left[y\left(t\right)\;y\left(t-\tau \right)\right]$, $f_{s}$ denotes the sampling frequency and E[·] denotes the expectation operator. The high resolution property of the AR spectral estimator (12) is mainly due to its ability to include all the autocorrelations appearing in the PSD (13) [20]. In fact, starting from (5), it is easy to show that the autocorrelation function $r_{y}\left(\tau \right)=r_{y}\left(-\tau \right)$ satisfies the relations

(14) $$\begin{array}{cc} r_{y}\left(0\right)+a_{1}\;r_{y}\left(1\right)+\cdots +a_{p}\;r_{y}\left(p\right) & =\sigma_{w}^{2} \end{array}$$

(15) $$\begin{array}{cc} r_{y}\left(\tau \right)+a_{1}\;r_{y}\left(\tau -1\right)+\cdots +a_{p}\;r_{y}\left(\tau -p\right) & =0,\;\forall \tau >0. \end{array}$$

This means that, if the p+1 autocorrelations $r_{y}\left(0\right),r_{y}\left(1\right),\cdots ,r_{y}\left(p\right)$ are available, all other autocorrelations can be recursively computed from (15).

The AR power spectral density (12) has proven to be a very effective tool for fault diagnosis [23,24,25,26,27,29,30]. Its high sensitivity to system changes, in fact, allows the definition of robust detection thresholds.

#### Determination of the Health Indicator

To extract a healthy indicator representing the current state of the system, we proposed a method based on the two main steps described in Figure 1 and Figure 2. As previously mentioned, the first phase involves the estimation of a suitable order *p* for the AR model. Different order selection criteria can be used, with the most popular being the Final Prediction Error criterion (FPE), the Akaike Information Criterion (AIC), and the Bayesian Information Criterion (BIC), also known as the Minimum Description Length (MDL) criterion. They are based on the minimization of different cost functions, which depend on the loss function $J(\hat{\theta })$ and on increasing values of the model order *p* [20,21,22].

During the second step (online CM), the health indicator (spectral distance), representing the state of the system, is continuously or periodically updated. The most popular spectral distances are the log-spectral distance, the Itakura–Saito spectral distance, and the Symmetric Itakura–Saito spectral distance (SISSD), also known as the COSH distance [40,41]. In this paper we adopt the SISSD distance:

(16) $$SISSD=\frac{1}{N_{f}}\;\sum_{i=1}^{N_{f}}\left(\frac{S_{AR}^{0}\left(f_{i}\right)}{S_{AR}\left(f_{i}\right)}-\log\frac{S_{AR}^{0}\left(f_{i}\right)}{S_{AR}\left(f_{i}\right)}+\frac{S_{AR}\left(f_{i}\right)}{S_{AR}^{0}\left(f_{i}\right)}-\log\frac{S_{AR}\left(f_{i}\right)}{S_{AR}^{0}\left(f_{i}\right)}-2\right)$$

where $N_{f}$ is the number of frequency points and $f_{i}\;\left(i=1,2,\cdots ,N_{f}\right)$ ranges from 0 to $f_{s}/2$. Since any wavelet detail y(t) is characterized by its specific frequency content, it is reasonable to select the frequency points in (16) according to the frequency bandwidth of y(t). In this way, it is possible to avoid using frequency points where the power of the compared signals is negligible. Therefore, for a given detail level *j*, the spectral distance (16) will change as follows:

(17) $$SISSD=\frac{1}{N_{f}^{j}}\;\sum_{i=1}^{N_{f}^{j}}\left(\frac{S_{AR}^{0}\left(f_{i},j\right)}{S_{AR}\left(f_{i},j\right)}-\log\frac{S_{AR}^{0}\left(f_{i},j\right)}{S_{AR}\left(f_{i},j\right)}+\frac{S_{AR}\left(f_{i},j\right)}{S_{AR}^{0}\left(f_{i},j\right)}-\log\frac{S_{AR}\left(f_{i},j\right)}{S_{AR}^{0}\left(f_{i},j\right)}-2\right)$$

where $S_{AR}^{0}\left(f,j\right),S_{AR}\left(f,j\right)$ are limited portions of the spectra $S_{AR}^{0}\left(f\right),S_{AR}\left(f\right)$:

(18) $$S_{AR}^{0}\left(f,j\right)=\left\{S_{AR}^{0}\left(f\right):f_{min}^{j}\leq f<f_{max}^{j}\right\}\;S_{AR}\left(f,j\right)=\left\{S_{AR}\left(f\right):f_{min}^{j}\leq f<f_{max}^{j}\right\}$$

with $f_{min}^{j}\geq 0$ and $f_{max}^{j}\leq fs/2$ chosen on the basis of the frequency content of the *j*-th detail.

The application of AR spectral estimation on short windows (data frames), within which the signal is assumed to be weakly stationary, is similar in spirit to the short-time Fourier transform; for this reason, it has also been called STAR (Short-Time AutoRegressive) [42,43]. It is worth mentioning that the STAR approach allows for quick detection of signal changes, making it suitable also for applications involving nonstationary signals [28,42,43,44,45].

## 3. Experimental Validation

To test the ability of the proposed method to detect and discriminate the presence of different faults, the effects of two different situations on the motor currents were considered: unbalanced load (case 1) and bearing faults (case 2). In the first case, experimental data were obtained with an in-house setup, while in the second case, freely available data from the repository [36] were used.

### 3.1. Case Study 1: Unbalanced Load

The proposed approach is tested on real data collected during the execution of electric cam motion tasks in a laboratory setup. This is a very common situation, as many industrial machines rely on electric cams to perform complex tasks that require synchronization among the various mechanisms involved [35].

#### 3.1.1. Experimental Setup

**Figure 3:**
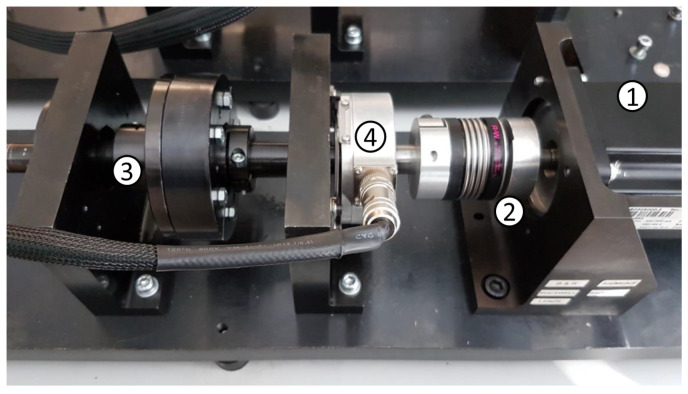
Experimental linear rigid mechanism setup. It is composed of the following: electrical motor (1), rigid joint (2), shaft and flywheel with two half-moon shaped weights (3), encoder (4).

**Figure 4:**
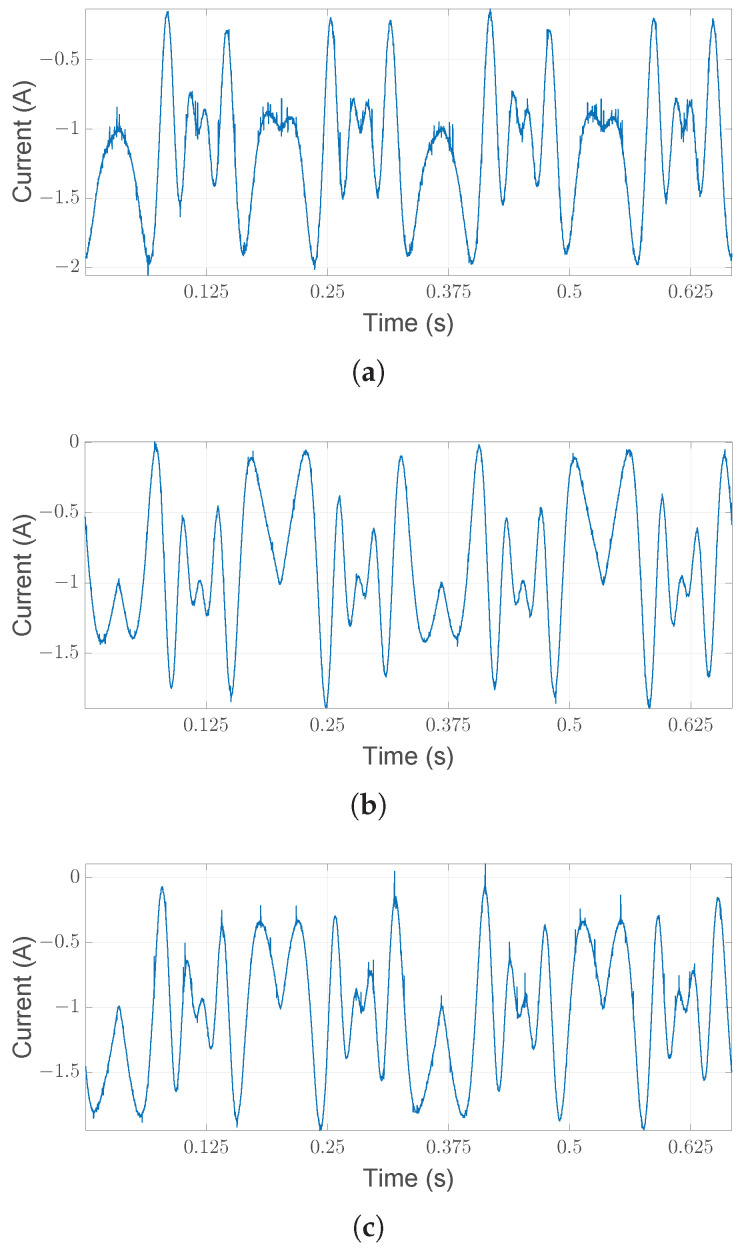
Measured motor currents (**a**–**c**). The impact of the electric cam on the three currents is evident from their respective shapes.

The experimental setup utilized is presented in Figure 3 and is composed of an electrical motor (1) that drives a shaft and flywheel with two half-moon-shaped weights (3) through a rigid joint (2). The cabled encoder (4) in the figure is not used in this experimental analysis and is not considered. The inertia of the system can be divided into two parts: one is fixed, with a value of $J_{fix}$ = 0.0015 kg/m^2^, and one is variable $J_{w}=J_{w_{1}}+J_{w_{2}}$ depending on the two attached weights, $J_{w_{1}}$ and $J_{w_{2}}$. The mechanism is driven by B&R equipment: the PLC is the *Automation PC 910* connected to an *ACOPOS P3* servo drive that controls a brushless motor *8LSA36DB030S000-3*. The nominal power of the motor is 848 W, the maximum speed is 9000 rpm and the maximum torque is 12 Nm. The acquisition system used to measure the currents is a National Instrument CompactDAQ (cDAQ-9189) with an NI-9215 module collecting three LEM LF-210S Hall-effect sensors at a sample rate of 25,600 Hz. The measurements are acquired in volts, and the conversion factor is 50 mV/A. Since the sampling frequency $f_{s}$ = 25,600 Hz is relatively high compared to the frequency range of interest in fault diagnosis of mechanical systems, the raw motor currents are decimated by a factor of 4 (via low-pass filtering and downsampling), resulting in $f_{s}$ = 6400 Hz. Decimation may also improve the accuracy of AR spectral estimation [46]. Figure 4 shows a section of the three motor currents after decimation.

**Figure 5:**
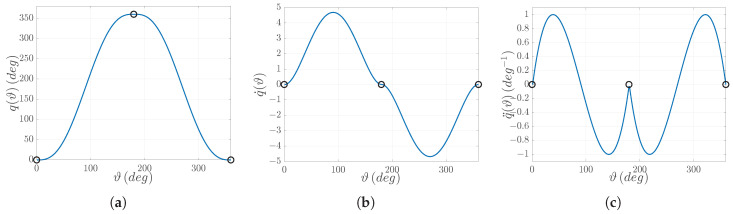
Piece-wise polynomial trajectories of the electric cam motion task: (**a**) Position q(ϑ); (**b**) Speed $\dot{q}\left(\vartheta \right)$; (**c**) Acceleration $\ddot{q}\left(\vartheta \right)$. Via-points are denoted by circles.

The system performs an electric cam as shown in Figure 5: a back-and-forth motion designed using piecewise fifth-order polynomials. It is worth recalling that electric cams are implemented by linking together the trajectories of the different motors involved in the synchronized task: a leader, known as the master, performs the guiding trajectory, while one or more followers, called slaves, move accordingly. The coupling is established geometrically so that any given master trajectory point ϑ(t) corresponds to a given slave trajectory point q(ϑ(t), denoted as q(ϑ) for simplicity. Typical implementations rely on the definition of via-points within the trajectory, connected through mathematical functions (polynomial functions in this case) that depend on the trajectory constraints. The constraints arise from the required trajectory derivatives at those via-points. In the present case, the virtual master, running at a constant speed of $\Omega_{p}$ = 1080°/s, is coupled with the slave, i.e., the electric motor shown in Figure 3. The synchronized motion task is defined through the following constraints:

(19) $$\begin{array}{cccccc} q_{1}\left(0{}^{\circ}\right) & =0{}^{\circ}, & q_{2}\left(180{}^{\circ}\right) & =360{}^{\circ}, & q_{3}\left(360{}^{\circ}\right) & =0{}^{\circ},\\ {\dot{q}}_{1}\left(0{}^{\circ}\right) & =0{}^{\circ}, & {\dot{q}}_{2}\left(180{}^{\circ}\right) & =0{}^{\circ}, & {\dot{q}}_{3}\left(360{}^{\circ}\right) & =0{}^{\circ},\\ {\ddot{q}}_{1}\left(0{}^{\circ}\right) & =0^{\circ^{-1}}, & {\ddot{q}}_{2}\left(180{}^{\circ}\right) & =0^{\circ^{-1}}, & {\ddot{q}}_{3}\left(360{}^{\circ}\right) & =0^{\circ^{-1}}, \end{array}$$

where $q_{i}\left(\vartheta \right),{\dot{q}}_{i}\left(\vartheta \right),{\ddot{q}}_{i}\left(\vartheta \right)$ denote the position, speed, and acceleration of the slave related to the *i*–th via-point.

To test the proposed monitoring approach, we sampled the slave drive currents $I_{1}\left(t\right),I_{2}\left(t\right),I_{3}\left(t\right)$ during the synchronized motion of the system with both symmetric and asymmetric (i.e., unbalanced) loads. The former is the healthy reference operating point, and the latter is the faulty one achieved by substituting one of the two half-moon-shaped weights with a slightly thicker or slightly thinner piece. In addition, in the symmetrical case, one of the two weights has been loosened by slightly unfastening the bolts that keep it in place to simulate a fault with increased degrees of severity. Those unbalanced loads mimicking incurring defects generate changes in the informative part of the currents $I_{1}\left(t\right),I_{2}\left(t\right)$, and $I_{3}\left(t\right)$ measurements, which in turn should be captured by the fault diagnosis procedure. The four tested configurations are reported below:

**Config.** **(1)** Symmetric load:
  (20) $$J_{w_{1}}=7.1305\cdot 10^{-4},\;J_{w_{2}}=7.1305\cdot 10^{-4}\;{\mathrm{kg}/m}^{2}$$
**Config.** **(2)** Asymmetric increased load:
  (21) $$J_{w_{1}}=7.1305\cdot 10^{-4},\;J_{w_{2}}=7.5030\cdot 10^{-4}\;{\mathrm{kg}/m}^{2}$$
**Config.** **(3)** Asymmetric decreased load:
  (22) $$J_{w_{1}}=7.1305\cdot 10^{-4},\;J_{w_{2}}=6.1725\cdot 10^{-4}\;{\mathrm{kg}/m}^{2}$$
**Config.** **(4)** Loose Symmetric load: same as Config. (1) but with loosened bolts in one of the weights:
  (23) $$\begin{array}{c} J_{w_{1}}=7.1305\cdot 10^{-4},\;J_{w_{2}}=7.1305\cdot 10^{-4}\;{\mathrm{kg}/m}^{2}\\ (\mathrm{loosened}\;\mathrm{bolts}) \end{array}$$

#### 3.1.2. Experimental Results

The setup and conditions have been explained in the previous sections. The analysis in Section 2.1.2 deals with providing the most appropriate Daubechies wavelet and detail level to identify different types of fault. As an example, Table 2 shows the differences in the entropy values $▵\varepsilon_{i,i+1}=\varepsilon_{i}-\varepsilon_{i+1}$ between two consecutive details $d_{i}^{k}\left(t\right)$ and $d_{i+1}^{k}\left(t\right)$. All values refer to the healthy current $I_{1}\left(t\right)$ and have been calculated using different Daubechies filters, starting at level j=2 and ending at level j=10. These values are correlated with the increase in regularity of the detail considered $d_{j}^{k}\left(t\right)$. As can be clearly seen in the Table, there is a significant change in behaviour between levels 5 and 6 (the most significant deviations are highlighted with bold values). Since the values obtained correlate with more regular behaviour and the details for higher values of *j* do not show significant differences, this indicates a remarkable regularity in the current trend. It is reasonable to assume that details at j<6 would not produce satisfactory results due to insufficient attenuation of the described effects with respect to the characteristics of interest. This conclusion is supported by the results discussed in Section 3.1.3. Similarly, using the details obtained from the wavelet analysis of the current $I_{1}\left(t\right)$ for levels j>6, similar results can be obtained (or even slightly better in the presence of certain perturbations) due to the lower presence of disturbances. The same analysis procedure was applied to the other healthy currents $I_{2}\left(t\right)$ and $I_{3}\left(t\right)$, obtaining similar results. The values shown in Table 2 suggest the most appropriate levels for analysis, while the type of wavelet filter can be chosen considering the filters that produce the highest values of entropy deviation. Table 3 shows these values for all the filters and currents considered. The values used in the following are highlighted in bold.

Based on the strategy of using filters that are not too long, the lowest order has been chosen, which gives the highest values of $▵\varepsilon_{i,i+1}$. It follows that in all subsequent steps of the proposed methodology, the details $d_{6}^{k}\left(t\right)$ and $d_{7}^{k}\left(t\right)$ of the db8 filter have been chosen as the most suitable to differentiate the different types of faults. Therefore, the currents $I_{1}\left(t\right)$, $I_{2}\left(t\right)$, and $I_{3}\left(t\right)$ have been processed with a wavelet filter db8, and the reconstructed details $d_{6}^{k}\left(t\right)$ and $d_{7}^{k}\left(t\right)$ have been chosen as starting data for the autoregressive spectral estimation process. All calculations were performed with MATLAB^TM^ version R2023b.

**Figure 6:**
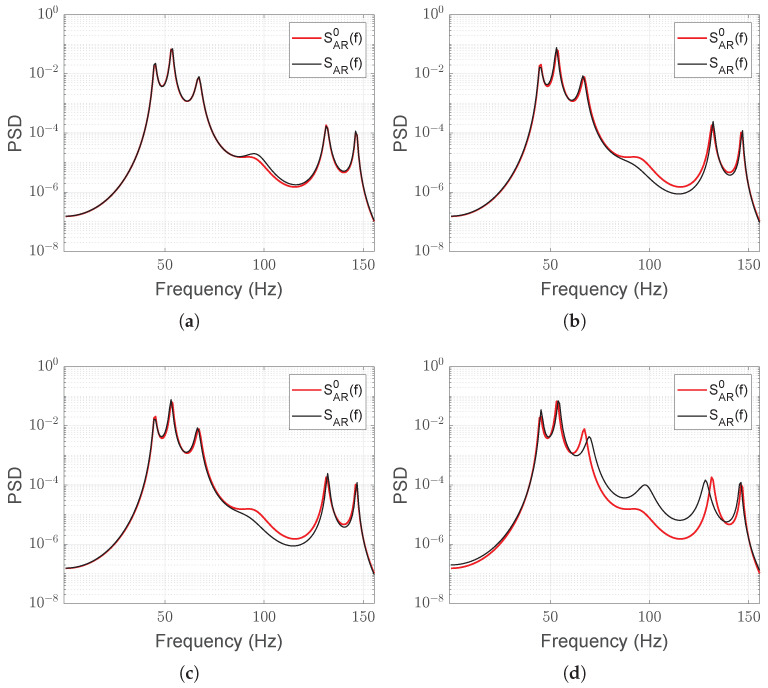
Comparison between the reference PSD $S_{AR}^{0}\left(f\right)$ and one of the PSDs $S_{AR}\left(f\right)$ obtained in the four possible health conditions: symmetric load (healthy state, (**a**)); symmetric increased load (**b**); asymmetric decreased load (**c**); loose symmetric load (**d**).

To compute the PSD of the nominal system $S_{AR}^{0}\left(f\right)$ as well as the PSDs $S_{AR}\left(f\right)$ of the system under the four possible health conditions, AR models have been estimated using the Least Squares algorithm on the reconstructed details $d_{6}^{k}\left(t\right)$ and $d_{7}^{k}\left(t\right)$ of the currents in configurations (1)–(4) (see Equations (20)–(23)). Each AR is of order p=150 and models a sequence of (*L* = 64,000) samples, obtained through a moving window with an overlap of 80% between consecutive frames. The AR order p=150 has been chosen by considering three popular order selection criteria: Final Prediction Error (FPE), Akaike Information Criterion (AIC), and Minimum Description Length (MDL) [20,21]. These methods involve minimizing order-dependent cost functions that include the sum of the squared prediction errors (7). Although none of the criteria exhibit a clear minimum, some of them tend to stabilize for orders *p* greater than 150. The order can be reduced of a few dozens while still maintaining fault detection and isolation, but with reduced robustness of the thresholds. The nominal PSD $S_{AR}^{0}\left(f\right)$ is the result of the mean of the spectra of the first ten AR ‘healthy’ models. Figure 6a–d illustrate the entity of the differences between $S_{AR}^{0}\left(f\right)$ and $S_{AR}\left(f\right)$ corresponding to a specific load condition. As expected, the nominal PSD and that obtained from a healthy model are close to indistinguishable, while as the severity of the fault increases, so does the divergence between the corresponding spectrum and $S_{AR}^{0}\left(f\right)$.

#### 3.1.3. Analysis of Results

**Figure 7:**
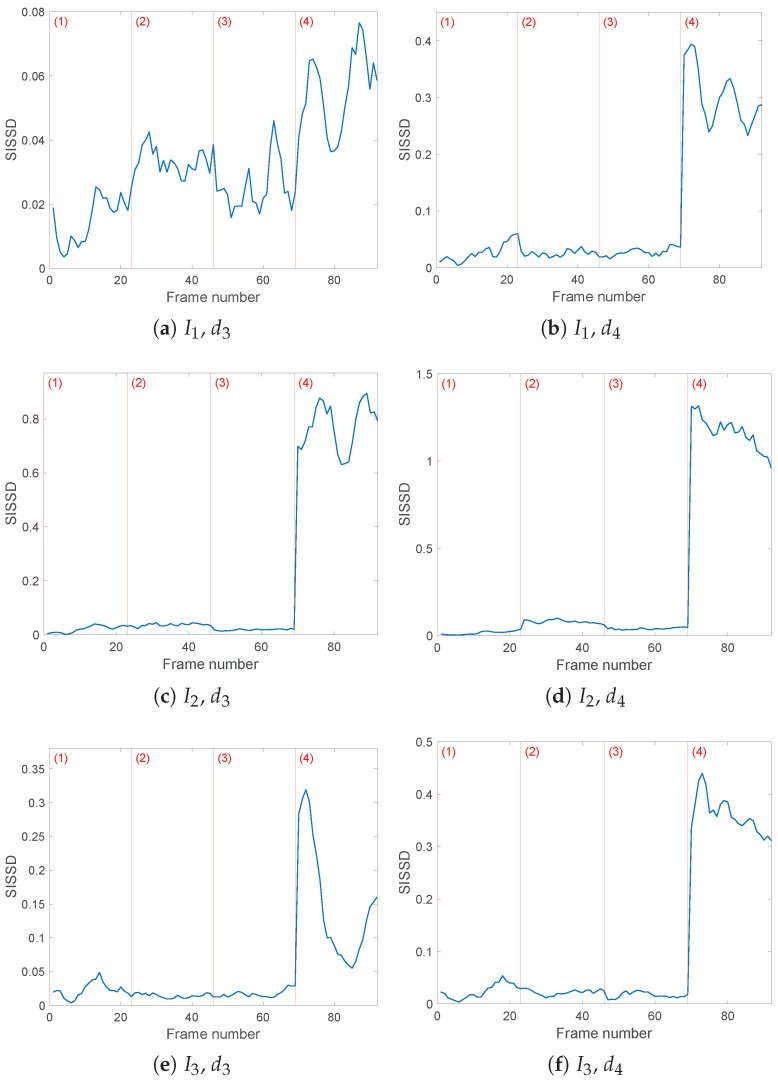
Evolution of the healthy indicator *SISSD* of the three currents in the four possible health conditions (1)–(4); wavelet decomposition was obtained through wavelet *db8* and three (**a**,**c**,**e**) or four (**b**,**d**,**f**) levels of details. *SISSD* values have been calculated starting from an AR model of order p=150 modeling a sequence of (*L* = 64,000) samples of the last reconstructed detail.

**Figure 8:**
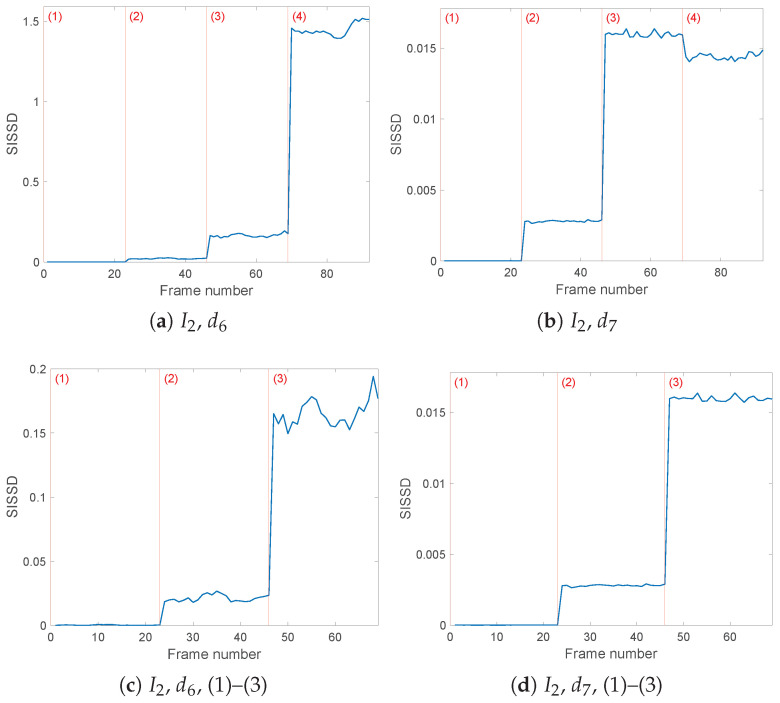
Evolution of the healthy indicator *SISSD* of Current 2, decomposed through wavelet *db8* and six (**a**,**c**) or seven (**b**,**d**) levels of details. *SISSD* values have been calculated starting from an AR model of order p=150 modeling a sequence of (*L* = 64,000) samples of the last reconstructed detail.

**Figure 9:**
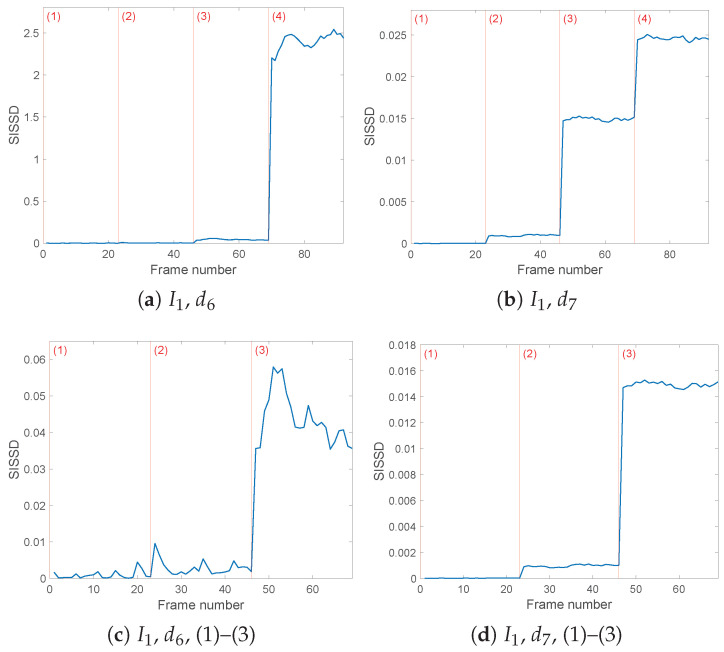
Evolution of the healthy indicator *SISSD* of Current 1, decomposed through wavelet *db8* and six (**a**,**c**) or seven (**b**,**d**) levels of details. *SISSD* values have been calculated starting from an AR model of order p=150 modeling a sequence of (*L* = 64,000) samples of the last reconstructed detail.

**Figure 10:**
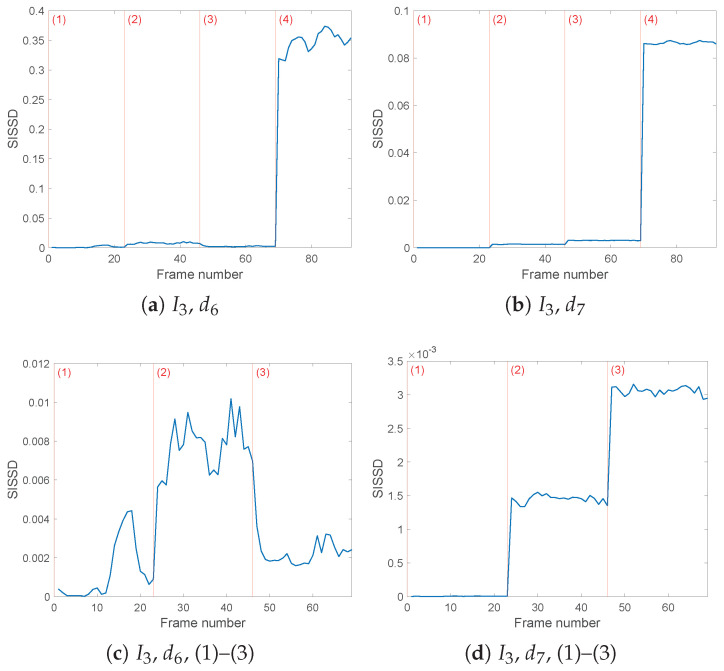
Evolution of the healthy indicator *SISSD* of Current 3, decomposed through wavelet *db8* and six (**a**,**c**) or seven (**b**,**d**) levels of details. *SISSD* values have been calculated starting from an AR model of order p=150 modeling a sequence of (*L* = 64,000) samples of the last reconstructed detail.

Each value of the SISSD distance in any of the Figure 7, Figure 8, Figure 9 and Figure 10 corresponds to the discrete spectral distance between frames of two spectra, the reference PSD and the PSD of a current sequence of *L* = 64,000 samples in one of the four load configurations. Of the entire spectrum, we consider only the bandwidth identified by the detail level taken into account. Figure 7 confirms the conclusions inferred by the entropy analysis by showing our inability to either detect or identify the faults in a simulated online monitoring phase if we rely on wavelet decomposition of detail level lower than 6. The favorable results, obtained with wavelet decomposition with j=6,7, are summarized in Table 4 for easier survey. It is possible to see how the faulty distances exceed those under healthy conditions by one order of magnitude.

Figure 7a,b show the evolution of the SISSD values considering current $I_{1}\left(t\right)$ values processed through a wavelet filter with the db8 wavelet and three and four detail levels. The severity of the third fault, consisting of loose symmetrical loads, is reflected in a visible increase of the spectral distance, a behavior that will be found in all of the exhibited results. With the exception of configuration (4), SISSD behaviour does not offer a quantifiable difference between healthy (1) and faulty (2)–(3) models. Currents $I_{2}\left(t\right),I_{3}\left(t\right)$ in Figure 7c–f report equivalent results.

The detail $d_{6}^{2}\left(t\right)$ of the db8 filter produces the best results for $I_{2}\left(t\right)$. Figure 8a shows that switching to configuration (4) causes a considerable increase of multiple orders of magnitude in the spectral distance values with respect to the healthy configuration (1). Figure 8c shows the smaller but nonetheless noticeable differences between configurations (1) and (2)–(3), as well as between the two less severe faults; the mean values of the healthy models are, as desired, close to zero with 0.0003, while once we consider faulty configurations, SISSD mean values increase to 0.0212 and 0.1655, respectively. The clear evolution of the health indicator throughout the four possible health conditions of the system is enough to detect and identify the faults from only this wavelet filter’s results, applied to $I_{2}\left(t\right)$ only. By combining this indicator with the information provided by the other two currents using the same preprocessing conditions, and the second set of results obtained from $d_{7}\left(t\right)$ detail, fault detection becomes even more robust.

The currents $I_{1}\left(t\right)$ and $I_{3}\left(t\right)$ produce similar results, with Figure 9a and Figure 10a showing the evolution of the health indicator computed from the details $d_{6}^{1}\left(t\right)$ and $d_{6}^{3}\left(t\right)$. As before, the magnitude of the third fault renders configuration (4) the easiest to detect. However, from Figure 9c, one can observe that from $I_{1}\left(t\right)$ models, the first fault, corresponding to an asymmetric increased load, is not straightforwardly detectable. Configuration (3) instead differs from the healthy conditions enough to bring the SISSD distance to increase from a mean of 0.009 to 0.0436. The third current leads to the least favourable results, with the healthy configuration models themselves diverging from the reference model, as visible from Figure 10c. The SISSD values between configurations (1) and (3) are too close to safely detect the asymmetric decreased load fault. Configuration (2) diverges more with respect to the healthy conditions, but other setup conditions produce a more conclusive output.

The db8 wavelet filter’s $d_{7}^{1}\left(t\right)$$d_{7}^{3}\left(t\right)$ details, according to the entropy analysis result, generate clearer and more performing outcomes. Not only Configuration (4), but also faulty cases (2) and (3) diverge from the healthy models in (1) and reference PSD; furthermore, the evolution of SISSD allows us to pick robust thresholds to identify the occurring fault among the three possible cases (Figure 9b and Figure 10b). As for current $I_{2}\left(t\right)$’s $d_{7}^{2}\left(t\right)$, similarly to the $d_{6}^{2}\left(t\right)$ outcome, the ‘healthy’ SISSD values are close to zero, and fault detection is ensured. This is the only case where, as visible in Figure 8b, configuration (3) models PSDs that are the furthest from the reference PSD. The loose symmetric load fault (3) and the asymmetric decreased loads (4) both lead to SISSD values around 0.015, with mean values being 0.0160 and 0.0145, respectively.

### 3.2. Case Study 2: Bearing Faults

#### 3.2.1. Experimental Setup

In order to further prove the efficacy of the proposed method, a second case study has been taken into consideration from the database [36]. This database offered current, temperature, vibration, and acoustic data of a rotating machine, measured under faulty conditions of various natures (unbalance, misalignment, and bearing faults), with a range of load options (either 0 Nm, 2 Nm, or 4 Nm). The rotating machine included a SIEMENS three-phase induction motor, driven at 380 V, 60 Hz, at a rated speed of 1770 rpm, increased to 3010 rpm for the collected data by a gearbox. A Valid Magnetic Ltd. hysteresis brake (*AHB-3A*) connected the simulated load to the machine, with a Datum Electronics torque meter (*M425*) to measure the load. The current data we have processed have been measured by four ceramic shear ICP-based accelerometers (*PCB35234*), “installed in the x- and y-directions of [the two] bearing housings. For a more detailed description of the testbed, we encourage the reading of the database accompanying article [36].

**Figure 11:**
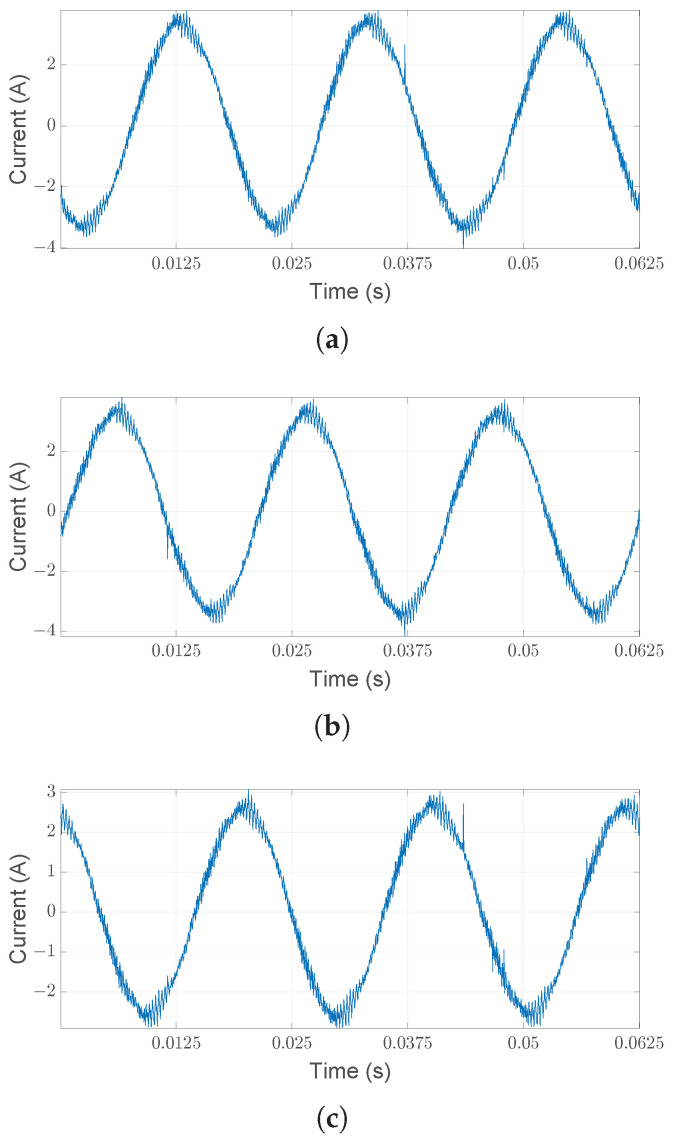
Measured motor currents (**a**–**c**) for the healthy case from [36].

Among the available data, we took into consideration the currents of three phases of bearing, measured under a 2 Nm load condition, in the following health conditions:

**Config.** **(1)** Healthy bearing;
**Config.** **(2)** Bearing with 0.3 mm inner race fault;
**Config.** **(3)** Bearing with 1.0 mm inner race fault;
**Config.** **(4)** Bearing with 3.0 mm inner race fault;

Similarly to the previous section, fault isolation concerns the same fault with different, increasing intensity, instead of multiple types of faults. The nature of the fault does not concern an unbalanced load, as was the case with the previous case study, but cracks to the inner race of the bearings. The chosen standardized NSK bearing (*NSK 6205 DDU*) has a ball diameter of 7.90 mm, a pitch diameter of 38.5 mm, a contact degree angle of zero degrees, and nine balls. Figure 11 shows a section of the three motor currents under normal operating conditions.

#### 3.2.2. Experimental Results and Analysis

**Figure 12:**
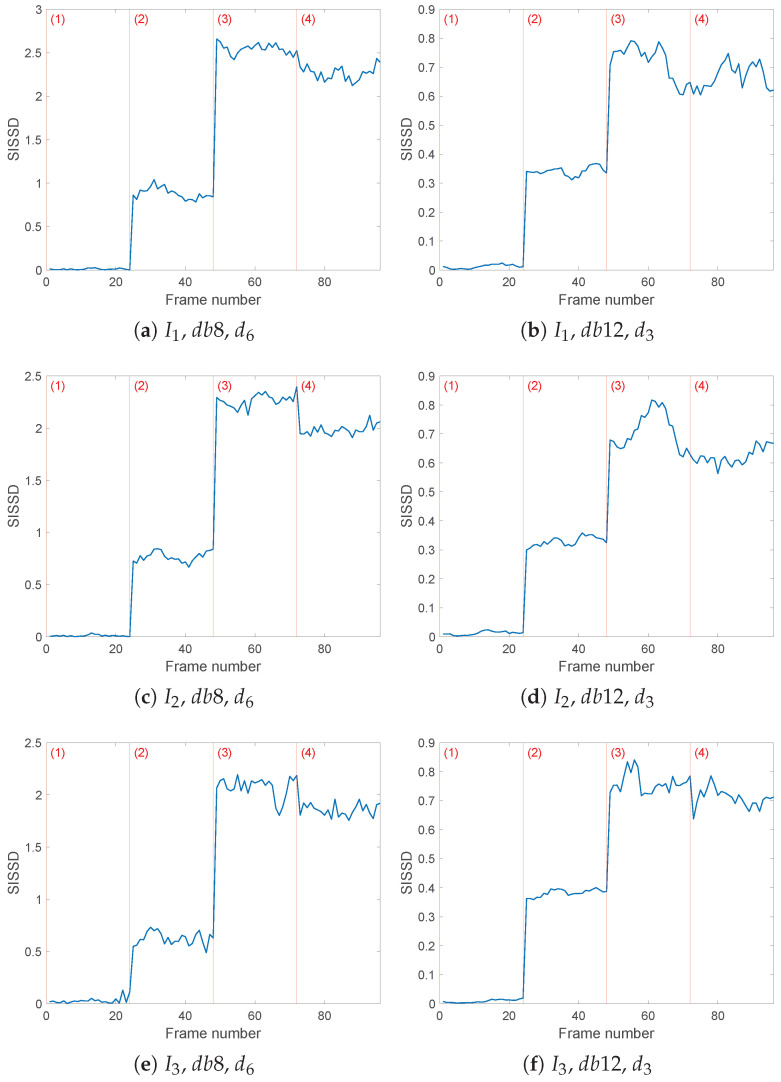
Evolution of the healthy indicator *SISSD* of the three currents in the four possible health conditions (1)–(4); wavelet decomposition was obtained through wavelet *db8* and six levels of details (**a**,**c**,**e**), or wavelet *db12* and three levels of details (**b**,**d**,**f**). *SISSD* values have been calculated starting from an AR model of order p=290 modeling a sequence of (*L* = 64,000) samples of the last reconstructed detail.

Also in this second case, the choice of wavelet filter dbM and analysis level *j* was made by evaluating the differences in entropy values $▵\varepsilon_{i,i+1}=\varepsilon_{i}-\varepsilon_{i+1}$ between two consecutive details $d_{i}^{k}\left(t\right)$ and $d_{i+1}^{k}\left(t\right)$. As an example, the results obtained for the current $I_{2}\left(t\right)$ are shown in Table 5 and Table 6. The choice of db12 and $d_{3}^{k}\left(t\right)$ gives the results in Figure 12 (right). From the values in Table 5, we also deduce as a possible alternative the use of db8 and $d_{6}^{k}\left(t\right)$, which gives similar results in Figure 12 (left).

Each frame of the SISSD spectral distance has been computed between the nominal PSD and the PSD of an AR model obtained during the phase of online condition monitoring. Every AR model is the Least Squares estimate of a window of *L* = 64,000 samples of the chosen reconstructed detail, with an order of p=290. Between consecutive windows, there is an overlap of 80% of the samples. The choice of the order has been guided by the Minimum Description Length (MDL) criterion, which for both configurations of the DWT processing returns that value as the minimum. The order can nonetheless be reduced while still maintaining fault detection and isolation, but with reduced threshold robustness.

The three currents lead to similar results, with fault detection always guaranteed for both of the chosen wavelet analysis configurations, either $db8,d_{6}^{k}$ or $db12,d_{3}^{k}$. In particular, switching from nominal behavior to the least intense fault, whose related distances are closest to the healthy values, nonetheless caused an increase of more than one order of magnitude in the health indicator. Table 7 summarizes the mean and standard deviation of the spectral distance values in the different health configurations for all three currents’ chosen details. It is possible to see how the third current’s $d_{6}^{3}\left(t\right)$ with the db8 wavelet filter leads to a SISSD between healthy AR models and the reference AR PSD of an average of 0.0301, the highest ‘healthy’ value. Despite this, fault detection is ensured with a percentage increase of 1978% up to 0.6255 for configuration (2), and even higher increases for more severe faults (3) and (4), whose mean SISSD values are 2.0755 and 1.8553, respectively.

Fault isolation proved more challenging; configuration (2), corresponding to the least severe fault of 0.1 mm to the inner race of the bearing, causes a noticeably smaller increase in the SISSD values than the other fault conditions (3) and (4). However, the two most severe simulated faults affect the health indicator similarly. In many cases, such as with details $db8,d_{6}^{2}\left(t\right)$ and $db8,d_{6}^{3}\left(t\right)$, as well as with all three currents considering wavelet db12 and $d_{3}^{k}\left(t\right)$, faulty configurations (3) and (4) cannot be robustly differentiated. To get a more robust fault isolation, we have to look at $I_{2}\left(t\right)$, processed through db8 with j=6: in this case the SISSD ranges from a mean of 0.0102 in healthy conditions, then increases to 0.7675±0.0491 in average if fault with intensity (2) occurs, and finally faulty configurations (3) and (4) cause the SISSD to rise again to mean values of 2.2665±0.0619 and 1.9833±0.0492 respectively. The considerations done in the previous section regarding the limits of the scalar nature of this health indicator can and should be considered here as well. Combining all three currents’ results and considering more than one wavelet filter can improve our chances of estimating the severity of the occurring fault, while detection is ensured with each of the proposed reconstructed wavelet details $d_{j}^{k}\left(t\right)$.

## 4. Conclusions

A novel motor current signature analysis approach has been proposed for fault diagnosis of mechanical systems driven by electric motors. The method exploits autoregressive spectral estimation and also takes advantage of the properties of the discrete wavelet transform. The proposed FD procedure is based on a health indicator, extracted from preprocessed current signals, that relies on the symmetric Itakura–Saito spectral distance. This indicator has proven to be highly sensitive to system changes, making it particularly effective for condition monitoring of electric motor-driven mechanisms. The FD procedure does not rely on any physical knowledge of the system and can be applied regardless of the type of component to be diagnosed (e.g., bearings, gears, shafts). Moreover, the steps involved in the procedure are not computationally demanding, making it suitable for implementation on edge-computing platforms and programmable logic controllers. The multiresolution analysis step, on the one hand, is performed using an efficient discrete-time algorithm based on a filter bank implementation to compute the wavelet details. On the other hand, highly efficient algorithms, such as recursive least squares and Burg’s method, are available for estimating autoregressive models. The effectiveness of the approach has been tested on real data collected from a laboratory setup during the execution of electric cam motion tasks, under unbalanced loads, as well as from a public data set measuring motor currents of a rotating machine with healthy and faulty bearings. The obtained results show the ability of the procedure in both fault detection and isolation. In particular, the chosen wavelet details always allow robust online fault detection, with at least one order of magnitude increase of the health indicator in fault conditions with respect to the healthy values. At its best, the health indicator of a single current’s detail is enough to perform fault isolation with robust thresholds. In cases where robust fault isolation proves more challenging for a scalar indicator, such as the one considered, the combination of multiple wavelet details of the available currents allows one to better identify the fault occurring or, in our case, gauge its severity. Future research will focus on applying the procedure to fault diagnosis in more complex scenarios. Among the most interesting future developments is the possibility of analyzing a partial reconstruction of currents according to (4) but obtained by considering only the details $d_{j}^{k}\left(t\right)$ of value levels close to those obtained from the analysis of healthy currents.

## Figures and Tables

**Table 1:** Nomenclature table.

Symbol	Meaning
$I_{1}\left(t\right),I_{2}\left(t\right),I_{3}\left(t\right)$	Three-phase motor current signals
$f_{s}$	Sampling frequency
dbM	Daubechies wavelet filter order *M*
*j*	Detail level of the wavelet analysis
$d_{j}^{k}\left(t\right)$	*j*–th detail of the wavelet analysis of the *k*–th current
$▵\varepsilon_{i,i+1}$	difference in entropy values between
	consecutive details $d_{i}\left(t\right)$ and $d_{i+1}\left(t\right)$
*p*	AR model order
*L*	Length of sequence modeled by the AR model
$S_{AR}^{0k}$	Reference AR power spectral density of the *k*-th current
$S_{AR}^{k}$	Power spectral density of an AR model of the *k*-th current
SISSD	Symmetric Itakura–Saito spectral distance

**Table 2:** Differences in entropy values between details at successive levels for different types of Daubechies filters applied to the healthy current $I_{1}\left(t\right)$.

	$▵\varepsilon_{9,10}$	$▵\varepsilon_{8,9}$	$▵\varepsilon_{7,8}$	$▵\varepsilon_{6,7}$	$▵\varepsilon_{5,6}$	$▵\varepsilon_{4,5}$	$▵\varepsilon_{3,4}$	$▵\varepsilon_{2,3}$	$▵\varepsilon_{1,2}$
db2	0.0170	0.0547	0.0562	0.1387	0.2335	0.1694	0.1914	0.0566	0.0169
db4	0.0120	0.0336	0.0518	0.1205	0.2923	0.1547	0.1269	0.0611	0.1644
db6	0.0118	0.0302	0.0516	0.0988	0.3243	0.0990	0.0754	0.2291	0.0921
db7	0.0114	0.0303	0.0509	0.0904	0.3654	0.0640	0.0995	0.2089	0.0983
**db8**	0.0122	0.0281	0.0513	0.0835	**0.3729**	0.0717	0.0348	0.3583	0.0111
db9	0.0114	0.0277	0.0516	0.0816	0.3649	0.0806	0.0355	0.3510	0.0254
db10	0.0118	0.0265	0.0532	0.0817	0.3643	0.0728	0.0723	0.2577	0.0521
db12	0.0126	0.0254	0.0536	0.0791	0.3710	0.0714	0.0393	0.3412	0.0722
**db20**	0.0124	0.0238	0.0553	0.0794	**0.3859**	0.0608	0.0407	0.3183	0.0986

**Table 3:** Maximum entropy differences $▵\varepsilon_{5,6}$ for all currents and different Daubechies filters.

	db2	db4	db6	db7	db8	db9	db10	db12	db20
$I_{1}$	0.2335	0.2923	0.3243	0.3654	**0.3729**	0.3649	0.3643	0.3710	0.3859
$I_{2}$	0.3066	0.2625	0.3142	0.3488	**0.4140**	0.3829	0.3514	0.4279	0.3926
$I_{3}$	0.2383	0.2727	0.3302	0.3337	**0.3492**	0.3418	0.3414	0.3539	0.3557

**Table 4:** Mean and standard deviation of the SISSD of the three currents in the four health configurations, with SISSD calculated through *db8* wavelet decomposition with 6 and 7 detail levels.

Detail		H	F1	F2	F3
$d_{6}$	$I_{1}$	0.0009±0.0011	0.0029±0.0020	0.0436±0.0069	2.4024±0.0938
	$I_{2}$	0.0003±0.0002	0.0212±0.0026	0.1655±0.0104	1.4470±0.0395
	$I_{3}$	0.0012±0.0015	0.0077±0.0013	0.0022±0.0006	0.3481±0.0164
$d_{7}$	$I_{1}$	1.4113 × 10^−5^ ± 8.9788 × 10^−6^	0.0010±0.0001	0.0149±0.0002	0.0246±0.0002
	$I_{2}$	5.9227 × 10^−6^ ± 4.2982 × 10^−6^	0.0028±0.0001	0.0160±0.0002	0.0145±0.0002
	$I_{3}$	4.5108 × 10^−6^ ± 2.4162 × 10^−6^	0.0015±0.0001	0.0031±0.0001	0.0864±0.0005

**Table 5:** Differences in entropy values between details at successive levels for different types of Daubechies filters applied to the healthy current $I_{2}\left(t\right)$.

	$▵\varepsilon_{9,10}$	$▵\varepsilon_{8,9}$	$▵\varepsilon_{7,8}$	$▵\varepsilon_{6,7}$	$▵\varepsilon_{5,6}$	$▵\varepsilon_{4,5}$	$▵\varepsilon_{3,4}$	$▵\varepsilon_{2,3}$	$▵\varepsilon_{1,2}$
db2	0.0255	0.0730	0.0108	0.2244	0.1180	0.1016	0.2647	0.2863	0.3095
db4	0.0156	0.0506	0.0108	0.2528	0.1134	0.1415	0.3444	0.3603	0.1513
db6	0.0143	0.0491	0.0143	0.2221	0.2333	0.0925	0.1763	0.3362	0.0899
db7	0.0147	0.0499	0.0174	0.2135	0.2671	0.0535	0.1537	0.3509	0.1461
db8	0.0132	0.0500	0.0216	0.1982	**0.3775**	0.0715	0.1030	0.3241	0.1257
db9	0.0136	0.0494	0.0276	0.1848	0.2854	0.0603	0.0994	0.4529	0.1132
db10	0.0139	0.0480	0.0359	0.1733	0.3236	0.0334	0.0709	0.5293	0.1317
db12	0.0138	0.0430	0.0479	0.1608	0.3030	0.0550	0.0567	**0.6028**	0.1431
db20	0.0152	0.0293	0.0609	0.1418	0.2501	0.1262	0.0209	0.5317	0.2536

**Table 6:** Maximum entropy differences $▵\varepsilon_{2,3}$ for all currents and different Daubechies filters.

	db2	db4	db6	db7	db8	db9	db10	db12	db20
$I_{1}$	0.3150	0.3352	0.3615	0.4272	0.4096	0.4780	0.4365	**0.5992**	0.6260
$I_{2}$	0.2863	0.3603	0.3362	0.3509	0.3241	0.4529	0.5293	**0.6028**	0.5317
$I_{3}$	0.3228	0.3395	0.3374	0.4144	0.5214	0.4529	0.4750	**0.5908**	0.6060

**Table 7:** Mean and standard deviation of the SISSD of the three currents in the four health configurations, with SISSD calculated through *db8* wavelet decomposition with 6 detail levels, or wavelet *db12* wavelet decomposition with 3 detail levels.

Detail		H	F1	F2	F3
db8, $d_{6}$	$I_{1}$	0.0105±0.0077	0.8811±0.0645	2.5462±0.0585	2.2648±0.0799
	$I_{2}$	0.0102±0.0083	0.7675±0.0491	2.2665±0.0619	1.9833±0.0492
	$I_{3}$	0.0301±0.0311	0.6255±0.0619	2.0755±0.1004	1.8553±0.0593
db12, $d_{3}$	$I_{1}$	0.0119±0.0067	0.3413±0.0148	0.7204±0.0593	0.6690±0.0431
	$I_{2}$	0.0124±0.0062	0.3294±0.0159	0.7118±0.0639	0.6227±0.0297
	$I_{3}$	0.0091±0.0052	0.3829±0.0121	0.7613±0.0345	0.7092±0.0315

## Data Availability

Data set available on request from the authors.
